# Fatigue in rheumatoid arthritis patients: The status, independent risk factors, and consistency of multiple scales

**DOI:** 10.1002/iid3.1313

**Published:** 2024-06-14

**Authors:** Jun Zhou, Wen Wang, Wenjia Gao, Yan Xu, Yinshan Zang

**Affiliations:** ^1^ Department of Rheumatology and Immunology The Affiliated Suqian First People's Hospital of Nanjing Medical University Suqian China

**Keywords:** arthritis, case–control studies, fatigue, rheumatoid, risk factors, surveys and questionnaires

## Abstract

**Introduction:**

Fatigue is a common symptom that negatively affects the outcomes and functions of rheumatoid arthritis (RA) patients. This study aimed to assess the fatigue by two scales and validate their consistency, also to comprehensively evaluate fatigue‐related risk factors in RA patients.

**Methods:**

In this case–control study, the fatigue of 160 RA patients and 60 healthy controls was evaluated by the Bristol Rheumatoid Arthritis Fatigue Multi‐Dimensional Questionnaire (BRAF‐MDQ) and the Chinese version of the Brief Fatigue Inventory (BFI‐C). The 28‐joint disease activity score using erythrocyte sedimentation rate of RA patients was assessed.

**Results:**

The BRAF‐MDQ and BFI‐C scores were elevated in RA patients versus healthy controls (all *p* < .001). Interestingly, BRAF‐MDQ global fatigue score positively correlated with BFI‐C global fatigue score in both RA patients (*r* = .669, *p* < .001) and healthy controls (*r* = .527, *p* < .001); meanwhile, Kendall's tau‐b test showed a high consistency between BRAF‐MDQ and BFI‐C global fatigue scores in RA patients (*W* = 0.759, *p* < .001) and healthy controls (*W* = 0.933, *p* < .001). Notably, higher education level (*В* = −4.547; 95% confidence interval: −7.065, −2.029; *p* < .001) and swollen joint count (*В* = 1.965; 95% confidence interval: 1.375, 2.554; *p* < .001) independently related to BRAF‐MDQ global fatigue score; higher education level (*В* = −0.613; 95% confidence interval: −0.956, −0.269; *p* = .001) and clinical disease activity index (*В* = 0.053; 95% confidence interval: 0.005, 0.102; *p* = .032) independently linked with BFI‐C global fatigue score.

**Conclusion:**

Fatigue commonly occurs in RA patients, which independently relates to education level and disease activity. Furthermore, BRAF‐MDQ and BFI‐C scales exhibit a high consistency in assessing fatigue.

## Graphical abstract

This study intended to assess fatigue by BRAF‐MDQ and BFI‐C scales, and validate their consistency, also to explore fatigue‐related risk factors in RA patients. Fatigue commonly occurs in RA patients, which independently relates to education level and disease activity. Furthermore, BRAF‐MDQ and BFI‐C scales exhibit a high consistency in assessing fatigue.

## 1 INTRODUCTION

Fatigue is an invasive and overwhelming symptom that is prevalent in rheumatoid arthritis (RA).^1^, ^2^, ^3^ Although fatigue in RA can be partially explained by inflammation, it may be more likely to be due to factors that are indirect to the disease, such as behavioral and psychological factors.^4^ Unlike normal fatigue, fatigue in RA cannot be improved with rest for patients, which has negatively affected their quality of life and caused obstacles to their mental, physical, employment, social, and other fields.^5^, ^6^, ^7^, ^8^ Therefore, exploring the fatigue condition and related risk factors may contribute to the management of fatigue and improve the prognosis of RA patients.

Previous studies have explored fatigue conditions and some fatigue‐related risk factors (including disease‐related, personal, social, and seasonal factors) in RA patients.^9^, ^10^, ^11^, ^12^, ^13^, ^14^ For example, one recent study shows that fatigue occurrence is 62%; meanwhile, education level and disease activity are related to fatigue score in RA patients.^9^ Simultaneously, another study indicates that some factors are associated with fatigue in RA patients, such as age, disease duration, functional disability, quality of life, and so on.^10^ Additionally, one previous study reveals that mental health and pain are important predictors of fatigue in RA patients.^11^ One study illustrates that RA patients experience greater fatigue during the winter.^12^ Moreover, some previous studies also exhibit that sleep disorders and comorbidities (such as hypertension, hypothyroidism, and deficiency anemias) are linked with fatigue in RA patients.^13^, ^14^ However, the gap of the above studies is that the vast majority of these studies use a single scale for fatigue evaluation in RA patients. More importantly, few studies verify the consistency among different scales for fatigue evaluation in RA patients.

The Bristol Rheumatoid Arthritis Fatigue Multi‐Dimensional Questionnaire (BRAF‐MDQ) is a fatigue assessment scale designed in collaboration with RA patients.^15^ Different from other scales, BRAF‐MDQ uniquely measures fatigue from four separate dimensions, which fully captures fatigue from the patient's perspective.^16^ Currently, BRAF‐MDQ has been widely used in clinical studies internationally.^17^ In addition, the Brief Fatigue Inventory (BFI) is also a fatigue assessment scale for RA patients, which is characterized by brevity and ease of understanding.^18^ The original BFI is translated into multiple languages, among which the Chinese version of the BFI (BFI‐C) provides a valid and reliable fatigue assessment instrument for Chinese RA patients.^18^ Considering the above advantages of BRAF‐MDQ and BFI‐C, we choose these two scales for fatigue assessments in RA patients. Meanwhile, to apply simple scales to replace complex scales for fatigue evaluation in clinical practice, our study also assessed the consistency between BRAF‐MDQ and BFI‐C scales.

Therefore, our study applied BRAF‐MDQ and BFI‐C scales to comprehensively evaluate fatigue and validate the consistency between scales; meanwhile, this study also aimed to assess the risk factors of fatigue in RA patients.

## 2 MATERIALS AND METHODS

### 2.1 Subjects

From December 2022 to February 2023, 160 RA patients treated in the Affiliated Suqian First People's Hospital of Nanjing Medical University were consecutively enrolled in this case–control study. The enrollment criteria contained the following: (i) diagnosis of RA per 1987 American College of Rheumatology (ACR) criteria or 2010 ACR/European League Against Rheumatism criteria^19^, ^20^; (ii) aged ≥ 18 years old; (iii) were voluntary for participation and signed the informed consent. The exclusion criteria contained the following: (i) had a prior history of malignancies, psychiatric diseases, neurological diseases, or major surgeries; (ii) had moderate‐to‐severe cognitive impairment to complete the questionnaire. Besides, 60 healthy subjects were enrolled as healthy controls with the following screening criteria: (i) had normal physical and biochemical indicators on physical examinations; (ii) aged ≥ 18 years old; (iii) had capable of accomplishing questionnaires; (iv) had no history of tumors, psychiatric diseases, neurological diseases, and major surgeries; and (v) volunteered to participate in the study and signed informed consent form. The Ethics Committee of the Affiliated Suqian First People's Hospital of Nanjing Medical University approved this study (approval number: 20230005).

### 2.2 Data collection

Clinical characteristics of RA patients were obtained after enrollment, which included (i) demographics: age, gender, body mass index (BMI), education level, marital status, employment status, and location (determined based on the information of patients’ identity cards); (ii) disease history: hypertension, hyperlipidemia, and diabetes; (iii) disease characteristics: disease duration, rheumatoid factor‐positive, anticitrullinated protein autoantibody‐positive, tender joint count (TJC), swollen joint count (SJC), erythrocyte sedimentation rate (ESR), C‐reactive protein, 28‐joint disease activity score using ESR (DAS28_ESR_ score), patient's global assessment score, physician's global assessment score, and clinical disease activity index (CDAI) score^21^, ^22^; (iv) treatment. Exposure variables included the clinical characteristics of RA patients (demographics, disease history, disease characteristics, and treatment).

### 2.3 Evaluation

Fatigue of RA patients and healthy controls were evaluated based on BRAF‐MDQ and BFI‐C after enrollment.^18^, ^23^ BRAF‐MDQ contains 20 items and encompasses a global score (Score 0–70) as well as physical (Score 0–22), living (Score 0–21), cognitive (Score 0–15), and emotional (Score 0–12) subdomain scores. The higher score indicates a more severe fatigue level. BFI‐C contains nine items and encompasses fatigue severity (mean value of three items) and fatigue interference (mean value of six items), and a global score is the mean value of nine items (Score 0–10). The higher score reflects the greater fatigue level. Functional disability and quality of life in RA patients were evaluated using Health Assessment Questionnaire Disability Index (HAQ) and short‐form health survey (SF12) scores, respectively.

### 2.4 Statistics

SPSS v22.0 (IBM Corp.) was adopted for analysis. GraphPad Prism v7.0 (GraphPad Software Inc.) was adopted for graph plotting. *T* test was utilized for group‐comparison analysis. Pearson's correlation test and Spearman correlation test were utilized for correlation analysis. Kendall's tau‐b test were utilized for consistency evaluation, and the consistency coefficient (*W*) > 0.7 was considered a good degree of consistency.^24^ Linear regression analysis was utilized for screening the factors related to fatigue. *p* < .05 was considered significant.

## 3 RESULTS

### 3.1 Baseline features of RA patients and healthy controls

The enrolled RA patients had a mean age of 57.9 ± 11.5 years with 44 (27.5%) males and 116 (72.5%) females. Meanwhile, healthy controls had a mean age of 48.5 ± 13.4 years with 22 (36.7%) males and 38 (63.3%) females. Notably, the mean age of RA patients was older than healthy controls (*p* < .001); meanwhile, there was a difference in education level between RA patients and healthy controls (*p* = .005). Moreover, there was no difference in gender (*p* = .186) or BMI (*p* = .146) between RA patients and healthy controls. More detailed characteristics were listed in Table 1.

**Table 1:** Clinical characteristics.

Items	RA patients (*N* = 160)	Healthy controls (*N* = 60)	*p*
Age (years), mean ± SD	57.9 ± 11.5	48.5 ± 13.4	<.001
Gender, no. (%)			.186
Male	44 (27.5)	22 (36.7)	
Female	116 (72.5)	38 (63.3)	
BMI (kg/m^2^), mean ± SD	23.3 ± 3.4	22.6 ± 2.8	.148
Education level, no. (%)			.005
Primary school or below	79 (49.4)	18 (30.0)	
Middle or high school	63 (39.4)	29 (48.3)	
Undergraduate or above	18 (11.3)	13 (21.7)	
Marital status, no. (%)			–
Married	136 (85.0)	–	
Single/divorced/widowed	24 (15.0)	–	
Employment status, no. (%)			–
Employed	82 (51.2)	–	
Unemployed	78 (48.8)	–	
Location, no. (%)			–
Urban	52 (32.5)	–	
Rural	108 (67.5)	–	
Hypertension, no. (%)	42 (26.3)	–	–
Hyperlipidemia, no. (%)	27 (16.9)	–	–
Diabetes, no. (%)	23 (14.4)	–	–
Disease duration (years), median (IQR)	8.0 (2.0–18.0)	–	–
RF positive, no. (%)	142 (88.8)	–	–
ACPA positive, no. (%)	146 (91.3)	–	–
TJC, median (IQR)	4.0 (3.0–6.0)	–	–
SJC, median (IQR)	3.0 (2.0–5.0)	–	–
ESR (mm/h), median (IQR)	51.9 (32.2–100.0)	–	–
CRP (mg/L), median (IQR)	24.1 (8.2–49.7)	–	–
DAS28_ESR_ score, median (IQR)	5.1 (4.3–5.5)	–	–
PGA score, median (IQR)	5.0 (4.0–6.0)	–	–
PhGA score, median (IQR)	5.0 (4.0–6.0)	–	–
CDAI score, median (IQR)	18.0 (14.3–22.0)	–	–
Treatment, no. (%)			–
NSAID	109 (68.1)	–	
GC	50 (31.3)	–	
csDMARD	148 (92.5)	–	
tsDMARD	30 (18.8)	–	
bDMARD	35 (21.9)	–	
Methotrexate administration, no. (%)	64 (40.0)	–	–
GC dosage, no. (%)			–
0 mg	110 (68.8)	–	
2.5 mg	8 (5.0)	–	
5 mg	39 (24.4)	–	
7.5 mg	1 (0.6)	–	
10 mg	2 (1.3)	–	

Abbreviations: ACPA, anticitrullinated protein autoantibody; bDMARDs, biologic disease‐modifying antirheumatic drugs; BMI, body mass index; CDAI, clinical disease activity index; CRP, C‐reactive protein; csDMARDs, conventional synthetic disease‐modifying antirheumatic drugs; DAS28_ESR_, 28‐joint disease activity score using ESR; ESR, erythrocyte sedimentation rate; GC, glucocorticoid; IQR, interquartile range; NSAID, nonsteroidal anti‐inflammatory drugs; PGA, patient's global assessment; PhGA, physician's global assessment; RA, rheumatoid arthritis; RF, rheumatoid factor; SJC, swollen joint count; TJC, tender joint count; tsDMARDs targeted synthetic disease‐modifying antirheumatic drugs.

In addition, the functional disability assessed by HAQ and quality of life evaluated by SF12 in RA patients were shown in Supporting Information S1: Table 1.

### 3.2 Comparison of BRAF‐MDQ and BFI‐C score between RA patients and healthy controls

**Figure 1:**
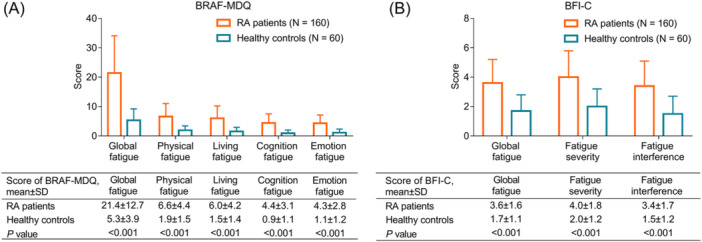
BRAF‐MDQ and BFI‐C score in RA patients and healthy controls. Comparison of BRAF‐MDQ score (A) and BFI‐C score (B) between RA patients and healthy controls. BRAF‐MDQ, Bristol Rheumatoid Arthritis Fatigue Multi‐Dimensional Questionnaire; BFI‐C, the Chinese version of the Brief Fatigue Inventory; RA, rheumatoid arthritis.

Regarding the BRAF‐MDQ score, the global fatigue score was elevated in RA patients compared with healthy controls (*p* < .001). Simultaneously, the physical fatigue score (*p* < .001), living fatigue score (*p* < .001), cognition fatigue score (*p* < .001), and emotion fatigue score (*p* < .001) were all increased in RA patients compared with healthy controls (Figure 1A).

In terms of the BFI‐C score, the global fatigue score was also increased in RA patients compared with healthy controls (*p* < .001). In addition, the fatigue severity score (*p* < .001) and fatigue interference score (*p* < .001) were both elevated in RA patients compared with healthy controls (Figure 1B).

### 3.3 Factors correlated with BRAF‐MDQ‐evaluated global fatigue score in RA patients

Univariate linear regression analysis exhibited that higher education level was related to lower global fatigue score evaluated by BRAF‐MDQ, while TJC, SJC, ESR, DAS28_ESR_ score, and CDAI score were correlated with higher global fatigue score evaluated by BRAF‐MDQ in RA patients. Further multivariate linear regression analysis revealed that higher education level was independently related to lower global fatigue score evaluated by BRAF‐MDQ (*В* = −4.547, *p* < .001), but SJC was independently related to higher global fatigue score evaluated by BRAF‐MDQ in RA patients (*В* = 1.965, *p* < .001) (Table 2).

**Table 2:** Linear regression analysis for global fatigue of BRAF‐MDQ.

Items	*В*	SE	95% CI		*t*	*p*
			Lower	Upper		
Univariate linear regression analysis						
Age (years)	0.087	0.088	−0.086	0.261	0.993	.322
Female	4.091	2.240	−0.333	8.515	1.826	.070
BMI (kg/m^2^)	−0.438	0.296	−1.023	0.147	−1.478	.141
Higher education level	−6.456	1.397	−9.216	−3.697	−4.621	<.001
Single/divorced/widowed	5.392	2.798	−0.134	10.918	1.927	.056
Unemployed	0.019	2.022	−3.975	4.012	0.009	.993
Rural	3.433	2.141	−0.795	7.661	1.604	.111
Hypertension	−2.510	2.288	−7.030	2.010	−1.097	.274
Hyperlipidemia	−3.303	2.686	−8.607	2.002	−1.230	.221
Diabetes	0.628	2.880	−5.061	6.317	0.218	.828
Disease duration (years)	0.090	0.104	−0.115	0.295	0.868	.387
RF positive	−0.266	3.199	−6.583	6.051	−0.083	.934
ACPA positive	6.438	3.540	−0.553	13.430	1.819	.071
TJC	2.079	0.322	1.443	2.715	6.456	<.001
SJC	2.207	0.301	1.612	2.803	7.323	<.001
ESR (mm/h)	0.083	0.026	0.032	0.133	3.200	.002
CRP (mg/L)	0.014	0.025	−0.036	0.063	0.534	.594
DAS28_ESR_ score	5.874	0.995	3.909	7.838	5.906	<.001
PGA score	−0.575	0.597	−1.755	0.605	−0.962	.337
PhGA score	−0.412	0.605	−1.608	0.783	−0.681	.497
CDAI score	0.745	0.143	0.464	1.027	5.230	<.001
NSAID treatment	−0.140	2.169	−4.424	4.143	−0.065	.948
GC treatment	1.287	2.178	−3.015	5.589	0.591	.555
csDMARD treatment	−0.405	3.837	−7.984	7.173	−0.106	.916
tsDMARD treatment	−1.077	2.588	−6.189	4.035	−0.416	.678
bDMARD treatment	−3.479	2.429	−8.277	1.319	−1.432	.154
Multivariate linear regression analysis						
Higher education level	−4.547	1.275	−7.065	−2.029	−3.567	<.001
SJC	1.965	0.299	1.375	2.554	6.578	<.001

*Note*: Variables in the univariate linear regression analysis were included in the multivariate linear regression analysis with the forward stepwise method.

Abbreviations: ACPA, anticitrullinated protein autoantibody; bDMARDs, biologic disease‐modifying antirheumatic drugs; BMI, body mass index; BRAF‐MDQ, Bristol Rheumatoid Arthritis Fatigue Multi‐Dimensional Questionnaire; CDAI, clinical disease activity index; CI, confidence interval; CRP, C‐reactive protein; csDMARDs, conventional synthetic disease‐modifying antirheumatic drugs; DAS28_ESR_, 28‐joint disease activity score using ESR; ESR, erythrocyte sedimentation rate; GC, glucocorticoid; NSAID, nonsteroidal anti‐inflammatory drugs; PGA, patient's global assessment; PhGA, physician's global assessment; RF, rheumatoid factor; SJC, swollen joint count; TJC, tender joint count; tsDMARDs targeted synthetic disease‐modifying antirheumatic drugs.

### 3.4 Factors correlated with BFI‐C‐evaluated global fatigue score in RA patients

Univariate linear regression analysis observed that higher education level and biologic disease‐modifying antirheumatic drugs treatment were associated with lower global fatigue score evaluated by BFI‐C, but the rural location, TJC, SJC, ESR, DAS28_ESR_ score, and CDAI score were linked with higher global fatigue score evaluated by BFI‐C in RA patients. Next, multivariate linear regression analysis showed that higher education level was independently correlated with lower global fatigue score evaluated by BFI‐C (*В* = −0.613, *p* = .001); however, CDAI score was independently related to higher global fatigue score evaluated by BFI‐C in RA patients (*В* = 0.053, *p* = .032) (Table 3).

**Table 3:** Linear regression analysis for global fatigue of BFI‐C.

Items	*B*	SE	95% CI		*t*	*p*
			Lower	Upper		
Univariate linear regression analysis						
Age (years)	0.016	0.011	−0.005	0.037	1.493	0.137
Female	0.139	0.278	−0.412	0.689	0.497	0.620
BMI (kg/m^2^)	−0.045	0.037	−0.117	0.027	−1.230	0.220
Higher education level	−0.735	0.174	−1.079	−0.392	−4.234	<0.001
Single/divorced/widowed	−0.283	0.348	−0.969	0.404	−0.813	0.418
Unemployed	0.082	0.249	−0.409	0.574	0.331	0.741
Rural	0.753	0.259	0.242	1.264	2.910	0.004
Hypertension	0.087	0.283	−0.471	0.645	0.308	0.759
Hyperlipidemia	−0.005	0.332	−0.662	0.651	−0.016	0.987
Diabetes	0.110	0.355	−0.591	0.810	0.310	0.757
Disease duration (years)	−0.003	0.013	−0.029	0.022	−0.270	0.788
RF positive	−0.482	0.392	−1.256	0.292	−1.229	0.221
ACPA positive	0.652	0.437	−0.212	1.516	1.491	0.138
TJC	0.196	0.042	0.114	0.278	4.696	<0.001
SJC	0.177	0.041	0.097	0.257	4.360	<0.001
ESR (mm/h)	0.011	0.003	0.005	0.017	3.540	0.001
CRP (mg/L)	0.001	0.003	−0.005	0.007	0.301	0.764
DAS28_ESR_ score	0.631	0.126	0.383	0.879	5.024	<0.001
PGA score	0.061	0.074	−0.084	0.207	0.831	.407
PhGA score	0.103	0.074	−0.044	0.250	1.389	.167
CDAI score	0.081	0.018	0.046	0.116	4.533	<.001
NSAID treatment	−0.399	0.265	−0.923	0.125	−1.504	.134
GC treatment	0.404	0.267	−0.123	0.930	1.514	.132
csDMARD treatment	−0.505	0.471	−1.435	0.424	−1.074	.285
tsDMARD treatment	−0.327	0.318	−0.955	0.300	−1.030	.305
bDMARD treatment	−0.679	0.296	−1.264	−0.094	−2.294	.023
Multivariate linear regression analysis						
Higher education level	−0.613	0.174	−0.956	−0.269	−3.523	.001
DAS28_ESR_ score	0.234	0.182	−0.125	0.594	1.287	.200
CDAI score	0.053	0.025	0.005	0.102	2.169	.032

*Note*: Variables in the univariate linear regression analysis were included in the multivariate linear regression analysis with the forward stepwise method.

Abbreviations: ACPA, anticitrullinated protein autoantibody; bDMARDs, biologic disease‐modifying anti‐rheumatic drugs; BFI‐C, the Chinese version of the Brief Fatigue Inventory; BMI, body mass index; CDAI, clinical disease activity index; CI, confidence interval; CRP, C‐reactive protein; csDMARDs, conventional synthetic disease‐modifying anti‐rheumatic drugs; DAS28_ESR_, 28‐joint disease activity score using ESR; ESR, erythrocyte sedimentation rate; GC, glucocorticoid; NSAID, nonsteroidal anti‐inflammatory drugs; PGA, patient's global assessment; PhGA, physician's global assessment; RF, rheumatoid factor; SJC, swollen joint count; TJC, tender joint count; tsDMARDs targeted synthetic disease‐modifying antirheumatic drugs.

### 3.5 Association of methotrexate administration with fatigue reflected by BRAF‐MDQ and BFI‐C in RA patients

There was no discrepancy in fatigue scores assessed by BRAF‐MDQ between RA patients treated with methotrexate administration and those without (all *p* > .05). In terms of fatigue scores assessed by BFI‐C, no difference was observed between RA patients treated with methotrexate administration and those without (all *p* > .05) (Table 4).

**Table 4:** Correlation of methotrexate administration with fatigue in RA patients.

Items	Methotrexate administration		*p*
	No (*n* = 96)	Yes (*n* = 64)	
Score of BRAF‐MDQ, mean ± SD			
Global fatigue	21.4 ± 12.8	21.3 ± 12.8	0.950
Physical fatigue	6.4 ± 4.5	7.0 ± 4.2	0.383
Living fatigue	6.1 ± 4.4	5.8 ± 3.8	0.587
Cognition fatigue	4.5 ± 3.0	4.3 ± 3.2	0.692
Emotion fatigue	4.4 ± 2.8	4.2 ± 2.7	0.683
Score of BFI‐C, mean ± SD			
Global fatigue	3.7 ± 1.6	3.6 ± 1.5	0.783
Fatigue severity	4.1 ± 1.8	3.9 ± 1.7	0.433
Fatigue interference	3.4 ± 1.7	3.4 ± 1.6	0.982

Abbreviations: BFI‐C, the Chinese version of the Brief Fatigue Inventory; BRAF‐MDQ, Bristol Rheumatoid Arthritis Fatigue Multi‐Dimensional Questionnaire; RA, rheumatoid arthritis.

### 3.6 Association of educational level with SJC and CDAI score in RA patients

The education level was negatively linked with SJC (*r* = −0.339, *p* < .001), but not correlated with CDAI score (*r* = −0.056, *p* = .480) in RA patients (Table 5). Additionally, in RA patients with SJC high (>3.0), the educational level was not linked with BRAF‐MDQ or BFI‐C scores (all *p* > .05). However, in RA patients with CDAI score high (>18.0), the educational level was inversely associated with BRAF‐MDQ and BFI‐C scores (all *p* < .05) (Table 6).

**Table 5:** Correlation of educational level with SJC and CDAI score in RA patients.

Items	*r*	*p*
SJC	−.339	<.001
CDAI score	−.056	.480

Abbreviations: CDAI, clinical disease activity index; RA, rheumatoid arthritis; SJC, swollen joint count.

**Table 6:** Correlation of educational level with fatigue in RA patients with high SJC or CDAI score.

Items	SJC high (>3.0)		CDAI score high (>18.0)	
	*r*	*p*	*r*	*p*
Score of BRAF‐MDQ				
Global fatigue	−.211	.070	−.418	<.001
Physical fatigue	−.195	.094	−.419	<.001
Living fatigue	−.150	.199	−.359	.002
Cognition fatigue	−.096	.414	−.328	.004
Emotion fatigue	−.166	.154	−.269	.019
Score of BFI‐C				
Global fatigue	−.117	.315	−.362	.001
Fatigue severity	−.127	.277	−.376	.001
Fatigue interference	−.083	.481	−.309	.007

Abbreviations: BFI‐C, the Chinese version of the Brief Fatigue Inventory; BRAF‐MDQ, Bristol Rheumatoid Arthritis Fatigue Multi‐Dimensional Questionnaire; CDAI, clinical disease activity index; RA, rheumatoid arthritis; SJC, swollen joint count.

### 3.7 Association of educational level with fatigue in healthy controls

Regarding the BRAF‐MDQ score, the educational level was inversely correlated with physical fatigue score (*p* = .001); however, it was not correlated with global fatigue, living fatigue, cognition fatigue, or emotion fatigue scores in healthy controls (all *p* > .05). Moreover, the educational level was not correlated with BFI‐C scores in healthy controls (all *p* > .05) (Supporting Information S1: Table 2). Univariate linear regression analysis showed that higher education level was not related to BRAF‐MDQ or BFI‐C scores in healthy controls (both *p* > .05) (Supporting Information S1: Table 3).

### 3.8 Correlation and consistency between BRAF‐MDQ and BFI‐C

**Figure 2:**
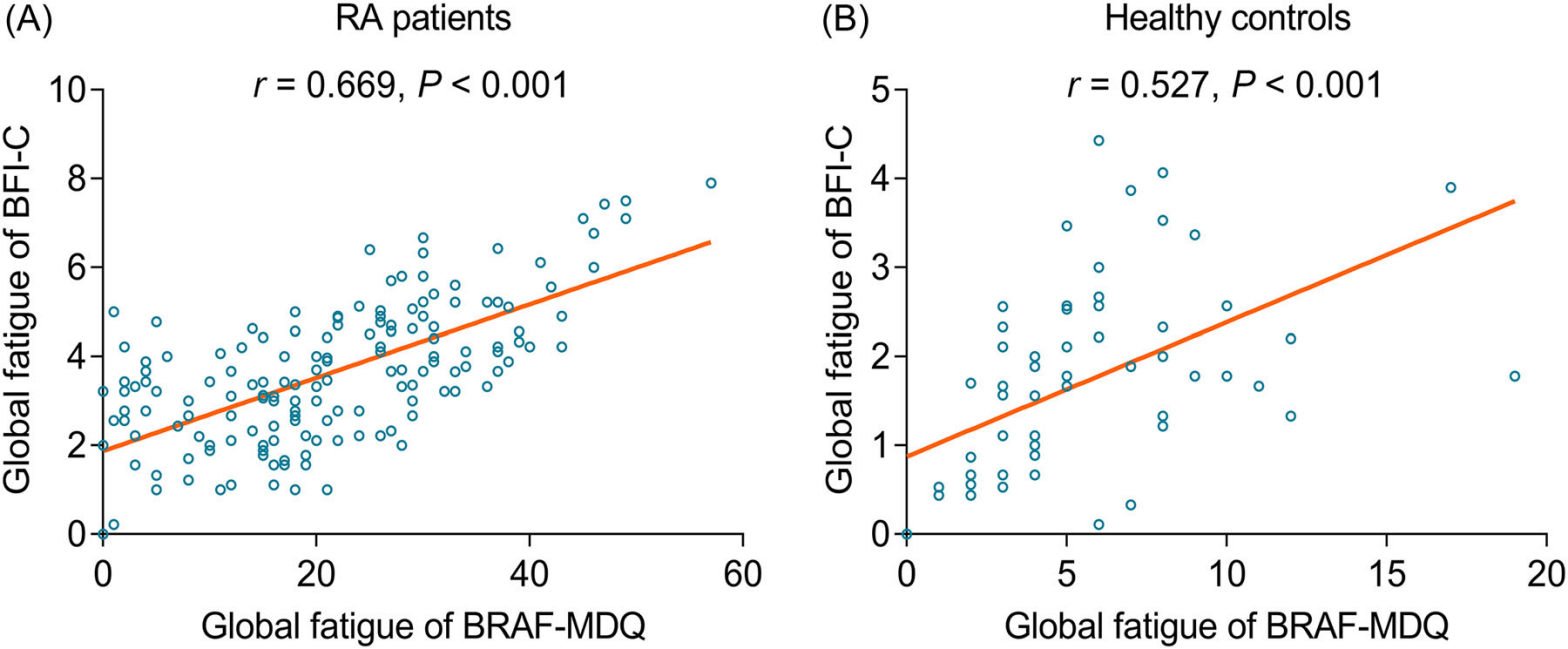
Correlation of BRAF‐MDQ with BFI‐C score in RA patients and healthy controls. The relationship between BRAF‐MDQ score and BFI‐C score in RA patients (A) and healthy controls (B). BFI‐C, the Chinese version of the Brief Fatigue Inventory; BRAF‐MDQ, Bristol Rheumatoid Arthritis Fatigue Multi‐Dimensional Questionnaire; RA, rheumatoid arthritis.

Notably, the global fatigue score evaluated by BRAF‐MDQ was positively associated with the global fatigue score assessed by BFI‐C in RA patients (*r* = .669, *p* < .001) (Figure 2A) and healthy controls (*r* = .527, *p* < .001) (Figure 2B). Furthermore, Kendall's tau‐b test indicated that there was a high consistency between BRAF‐MDQ and BFI‐C in RA patients (*W* = 0.759, *p* < .001) and healthy controls (*W* = 0.933, *p* < .001).

## 4 DISCUSSION

The main findings of our study were as follows: (1) According to the BRAF‐MDQ score, the global fatigue score, the physical fatigue score, living fatigue score, cognition fatigue score, and emotion fatigue score were increased in RA patients compared with healthy controls. (2) Based on the BFI‐C score, the global fatigue score, the fatigue severity score, and fatigue interference score were elevated in RA patients compared with healthy controls. (3) Higher education level and SJC independently related to BRAF‐MDQ global fatigue score; higher education level and CDAI independently linked with BFI‐C global fatigue score in RA patients. (4) There was a high consistency between BRAF‐MDQ and BFI‐C global fatigue scores in RA patients and healthy controls.

Although the exact mechanism is still unclear, evidence shows that disease‐related factors (inflammation, pain, and so on), medication administration, cognitive and behavioral factors, and personal factors (personal responsibilities, personal environments, social support, personal health, and dietary patterns) can contribute to fatigue in RA patients.^4^, ^25^, ^26^, ^27^ As a common chronic symptom in RA patients, fatigue brings great burdens to patients, including daily activity obstacles, decreased work ability, economic loss, and so on.^28^, ^29^ Previous studies show the prevalence of fatigue in RA is about 44.00%–71.25% and the fatigue score in RA patients is higher than in healthy controls, indicating that fatigue is widespread among RA patients.^30^, ^31^, ^32^ Similar to these studies, our study presented that the fatigue scores reflected by BRAF‐MDQ and BFI‐C scale were increased in RA patients compared with healthy controls. The possible reasons for fatigue in RA patients were as follows: (1) RA patients had high levels of inflammation, which was considered to be positively correlated with fatigue.^25^, ^33^ (2) RA patients had reduced self‐efficacy, low mood, poor sleep at night, and so on, which might also induce fatigue.^26^ Therefore, the fatigue score was higher in RA patients.

Exploring the risk factors of fatigue for early management is of great significance for the treatment of RA patients. One previous study shows that female sex, functional capacity, physical pain, mental health, and disease activity are risk factors for fatigue in RA patients.^32^ Furthermore, one study finds that pain, morning stiffness, hemoglobin, joint tenderness, and disease activity are associated with fatigue in RA patients.^6^ Another study exhibits that age, education, hypertension, disease activity, and other factors are correlated with fatigue in RA patients.^9^ Our study found that education level and disease activity were independent risk factors of fatigue reflected by the BRAF‐MDQ and BFI‐C scales. The possible explanations were as follows: (1) RA patients with a high education level might have a better understanding of the disease and good medication adherence^34^; meanwhile, they received more social support when facing disease symptoms, which was considered to be imperative in coping with fatigue.^35^, ^36^ The above advantages might alleviate fatigue symptoms. Furthermore, RA patients with a low education level might be more inclined to work in manual jobs, which increased their fatigue.^37^ (2) Disease activity reflected the disease status. RA patients with high disease activity might have additional joint pain and elevated inflammation, which caused severe fatigue.^6^, ^31^, ^38^ (3) Our study used BRAF‐MDQ and BFI‐C scales to assess fatigue, which was different from scales used in other previous studies.^6^, ^9^, ^32^ Moreover, differences in the included population between our study and other previous studies might cause different results. Therefore, there might be some differences in factors related to fatigue between our study and other previous studies. Notably, there was a difference in age between RA patients and healthy controls in our study, thus our study further assessed whether age was related to fatigue in RA patients. The result suggested that age was not independently related to global fatigue score evaluated by BRAF‐MDQ or by BFI‐C in RA patients. The finding of our study indicated that fatigue in RA patients might be caused by the disease itself rather than age. Moreover, there was a difference in education level between RA patients and healthy controls in our study. To further explore whether the correlation between education level and fatigue is universal or RA‐specific, our study investigated the correlation of education level with fatigue in healthy controls and found a very weak correlation. Meanwhile, higher education level was not an independent risk factor of fatigue reflected by the BRAF‐MDQ and BFI‐C scales in healthy controls. This finding indicated that the correlation between education level and fatigue was RA‐specific.

Interestingly, our study found that risk factors associated with fatigue in RA patients were slightly different between the BRAF‐MDQ scale and the BFI‐C scale. The risk factors for fatigue in RA patients assessed by the BRAF‐MDQ scale were higher education level and SJC, while the risk factors for fatigue in RA patients evaluated by the BFI‐C scale were high education and SJC. The difference between the two scales might be due to their different modes of questioning RA patients: The BRAF‐MDQ scale consulted RA patients in detail whether their living conditions were affected by fatigue.^7^ However, the BFI‐C scale directly asked RA patients to score for fatigue.^18^ Thus, it was hypothesized that different modes of questioning patients from the two scales might have a certain influence on the risk factor assessment of fatigue in RA patients. Nevertheless, further verification was required for this hypothesis.

The current drugs for RA mainly include nonsteroidal anti‐inflammatory drugs, glucocorticoids, and DMARDs.^39^ Notably, some studies exhibit that the use of DMARDs alleviates fatigue compared to placebo in RA patients.^40^, ^41^ However, there is no difference in the effect of different drugs on fatigue in RA patients.^25^, ^42^ For example, one previous study shows that the type of medicine is not related to fatigue in RA patients.^42^ Another study also illustrates that different drug treatments did not affect fatigue severity in RA patients.^25^ The result of our study was similar to the findings of these above studies,^25^, ^42^ which revealed that specific drug regimens were not independently linked with fatigue in RA patients.

In addition, due to the different evaluation emphasis of each scale, there may be some discrepancies in fatigue assessment when using diverse scales in RA patients.^43^ However, the consistency among different scales in assessing fatigue is not evaluated in the previous studies.^9^, ^10^, ^11^ Therefore, our study included the BRAF‐MDQ scale and BFI‐C scale, then confirmed their good consistency in assessing fatigue scores in RA patients. Considering that it is more convenient to use the BFI‐C scale than the BRAF‐MDQ scale,^18^, ^44^ the BFI‐C scale could be considered as a substitute of the BRAF‐MDQ scale to evaluate the fatigue score in RA in clinical practice.

Notably, there are many different scales to evaluate fatigue, while few studies use multiple scales for fatigue evaluation and verify their consistency. Our study evaluated fatigue by two scales (the BRAF‐MDQ and the BFI‐C scales), and then validated their consistency in RA patients. The result of our study showed that the BRAF‐MDQ and the BFI‐C scales exhibited a high consistency in the clinical assessment of fatigue in RA patients, which was not reported in previous studies. Based on the results of our study, future studies should consider expanding the sample size or longitudinally assessing the fatigue of RA patients for further verification.

There were several limitations in this study: (1) The number mismatch between RA patients and healthy controls might interfere with the statistical effect. (2) Although the sample size in our study was relatively large (*N* = 160), further studies should consider including an even greater sample size to obtain a clearer conclusion. (3) Our study was cross‐sectional, and longitudinal studies with repeat measures are needed to further explore risk factors of fatigue. (4) Our study was performed in a single center in China, and the results might not be applicable to the general population.

In conclusion, fatigue is prevalent and its risk factors include low education level and high disease activity in RA patients. Fatigue in RA patients is not influenced by different medical treatments for RA. Moreover, fatigue assessment by BFI‐C and BRAF‐MDQ scales are interchangeable.

## AUTHOR CONTRIBUTIONS

Jun Zhou, Wen Wang, and Yinshan Zang contributed to conception and design of the study. Wenjia Gao and Yan Xu contributed to data acquisition. Jun Zhou and Yinshan Zang analyzed the data. Jun Zhou, Wen Wang, Wenjia Gao, Yan Xu, and Yinshan Zang drafted and revised the manuscript. All authors have read and approved the final manuscript.

## CONFLICT OF INTEREST STATEMENT

The authors declare no conflict of interest.

## ETHICS STATEMENT

Participants signed informed consent form. The study had the approval of the Ethics Committee.

## Supporting information

Supporting Information

## Data Availability

The data that support the findings of this study are available from the corresponding author upon reasonable request.
