# Supplementary material for: Fatigue in rheumatoid arthritis patients: The status, independent risk factors, and consistency of multiple scales
Source: Immun Inflamm Dis. 2024 Jun 14;12(6):e1313. doi: 10.1002/iid3.1313 (PMC11177286; doi:10.1002/iid3.1313)
Supplement: Supplementary file 1 — Supporting Information [file IID3-12-e1313-s001.docx]

**SUPPLEMENTARY TABLE 1** Functional disability and quality of life in RA patients.

| Items | RA patients (N = 160) |
| --- | --- |
| HAQ score, mean±SD | 19.9±12.3 |
| SF12 score, mean±SD | 572.8±166.7 |

RA, rheumatoid arthritis; HAQ, Health Assessment Questionnaire Disability Index; SD, standard deviation; SF12, short-form health survey.

**SUPPLEMENTARY TABLE 2**. Correlation between fatigue and educational level in healthy controls.

| Items | *ρ* value | *P* value |
| --- | --- | --- |
| BRAF-MDQ |  |  |
| Global fatigue | -0.165 | 0.207 |
| Physical fatigue | -0.411 | 0.001 |
| Living fatigue | -0.005 | 0.969 |
| Cognition fatigue | 0.042 | 0.752 |
| Emotion fatigue | -0.015 | 0.908 |
| BFI-C |  |  |
| Global fatigue | 0.033 | 0.802 |
| Fatigue severity | -0.014 | 0.915 |
| Fatigue interference | 0.054 | 0.682 |

BRAF-MDQ, Bristol Rheumatoid Arthritis Fatigue Multi-Dimensional Questionnaire; BFI-C, the Chinese version of the Brief Fatigue Inventory.

**SUPPLEMENTARY TABLE 3**. Linear regression analysis for global fatigue of BRAF-MDQ and BFI-C in healthy controls.

| Items | *B* | *SE* | 95%CI | | *t* value | *P* value |
| --- | --- | --- | --- | --- | --- | --- |
|  |  |  | Lower | Upper |  |  |
| **Univariate linear regression analysis for global fatigue of BRAF-MDQ** | | | | | | |
| Higher education level | -0.567 | 0.703 | -1.975 | 0.841 | -0.806 | 0.424 |
| **Univariate linear regression analysis for global fatigue of BFI-C** | | | | | | |
| Higher education level | 0.078 | 0.203 | -0.327 | 0.484 | 0.386 | 0.701 |

BRAF-MDQ, Bristol Rheumatoid Arthritis Fatigue Multi-Dimensional Questionnaire; BFI-C, the Chinese version of the Brief Fatigue Inventory.
